# Metabolic health's central role in chronic kidney disease progression: a 20-year study of obesity-metabolic phenotype transitions

**DOI:** 10.1038/s41598-024-56061-x

**Published:** 2024-03-04

**Authors:** Shayesteh Khalili, Seyed Amir Ahmad Safavi-Naini, Paniz Zarand, Safdar Masoumi, Yeganeh Farsi, Farhad Hosseinpanah, Fereidoun Azizi

**Affiliations:** 1 Department of Internal Medicine, School of Medicine, Imam Hossein Hospital, Shahid Beheshti University of Medical Sciences, Tehran, Iran; 2 Research Institute for Gastroenterology and Liver Diseases, Shahid Beheshti University of Medical Sciences, Tehran, Iran; 3 Endocrine Research Center, Research Institute for Endocrine Sciences, Shahid Beheshti University of Medical Sciences, Tehran, Iran; 4 Obesity Research Center, Research Institute for Endocrine Sciences, Shahid Beheshti University of Medical Sciences, No. 23, Parvaneh StreetVelenjak, P.O. Box: 19395-4763, Tehran, 19395-4763 Iran

**Keywords:** Endocrinology, Nephrology

## Abstract

This study investigates the risk of chronic kidney disease (CKD) across four metabolic phenotypes: Metabolically Healthy-No Obesity (MH-NO), Metabolically Unhealthy-No obesity (MU-NO), Metabolically Healthy-Obesity (MH-O), and Metabolically Unhealthy-Obesity (MU-O). Data from the Tehran Lipid and Glucose Study, collected from 1999 to 2020, were used to categorize participants based on a BMI ≥ 30 kg/m^2^ and metabolic health status, defined by the presence of three or four of the following components: high blood pressure, elevated triglycerides, low high-density lipoprotein, and high fasting blood sugar. CKD, characterized by a glomerular filtration rate < 60 ml/min/1.72 m^2^. The hazard ratio (HR) of CKD risk was evaluated using Cox proportional hazard models. The study included 8731 participants, with an average age of 39.93 years, and identified 734 incidents of CKD. After adjusting for covariates, the MU-O group demonstrated the highest risk of CKD progression (HR 1.42–1.87), followed by the MU-NO group (HR 1.33–1.67), and the MH-O group (HR 1.18–1.54). Persistent MU-NO and MU-O posed the highest CKD risk compared to transitional states, highlighting the significance of exposure during early adulthood. These findings emphasize the independent contributions of excess weight and metabolic health, along with its components, to CKD risk. Therefore, preventive strategies should prioritize interventions during early-adulthood.

## Introduction

Chronic kidney disease (CKD) is a major public health concern that affects approximately 700 million people worldwide^1^. CKD is marked by a gradual decline in renal function and impaired blood filtration. As the disease progresses, it can lead to various complications in other organs, including anemia, bone disorders, and cardiovascular diseases (CVD)^2^. The primary causes of CKD are underlying conditions that gradually damage the kidneys over time. The most common predisposing factors for CKD include Diabetes Mellitus (DM), hypertension, a family history of CKD, heart diseases, and obesity^2,3^.

Amidst these risk factors, many, such as hypertension, DM, excess weight, and dyslipidemia, are also recognized as integral components of Metabolic Syndrome (MetS)^4^. In recent years, scholarly debates have intensified regarding the complex interactions between obesity and metabolic health, especially in relation to their collective impact on CVD and CKD^5,6^. A comprehensive understanding of these components' definition, epidemiology, and associated risk factors is crucial for demystifying the intricate nexus they form.

Obesity is a global public health issue^7^, and current trends suggest that more than half of the world’s population will have obesity by 2030. Excess weight can adversely affect any human organ, leading to cardiovascular, psychological, neoplastic, hepatic, and inflammatory diseases^8,9^. Exposure to obesity, especially from early adulthood, can also lead to DM, hypertension, and dyslipidaemia^8,9^. Moreover, obesity is a common risk factor for kidney diseases, increasing the risk of CKD by 40%^10^. Various definitions of obesity, based on Body Mass Index (BMI) or waist circumference (WC), have been employed, all converging on similar conclusions regarding its association with CKD^11,12^.

Parallel to obesity, MetS has emerged as a formidable risk factor for a myriad of diseases, affecting one in five individuals globally. This syndrome, defined by metabolic abnormalities such as central obesity, hypertension, elevated triglycerides, reduced High-Density Lipoprotein (HDL), and high blood glucose, independently heightens the risk for diseases including CKD^5,13^. While the initial proposal of MetS sparked debates over its cumulative risk impact on CVD and CKD, contemporary consensus leans towards recognizing a synergistic effect among its components^14,15^.

In this evolving landscape, the concept of metabolic health has garnered attention, offering a holistic view of the body’s metabolic processes and overall physiological health^15^. This emerging paradigm appreciates the complex interplay among diverse metabolic functions and their cumulative impact on health, extending beyond traditional metrics like weight indices^13^.

Recent studies have identified a unique subset of individuals with obesity characterized by a low burden of metabolic abnormalities, termed Metabolically Healthy Obesity (MHO). Studies suggest individuals with MHO have better glucose resistance, blood pressure, lipid profile, and inflammatory conditions than metabolically unhealthy individuals with obesity^2,13^. However, investigations into the long-term clinical outcomes associated with MHO have yielded conflicting results, underscoring the necessity for further research, particularly on phenotype transitions^5,16,17^.

To effectively tailor interventions, it is imperative to unravel the dynamics and temporal impact of predisposing factors on CKD incidence. Yet, only a limited number of studies have explored the time-variant interplay among obesity, metabolic health, and CKD incidence^18^. Addressing this gap, our study delves into the CKD risk associated with four distinct phenotypes of metabolic health states and obesity: Metabolically Healthy-No Obesity (MH-NO), MHO, Metabolically Unhealthy-No Obesity (MU-NO), and Metabolically Unhealthy Obesity (MU-O). We also scrutinize the CKD risk in individuals undergoing phenotypic transitions over time, thereby contributing to a deeper understanding of CKD's multifactorial nature.

## Material and methods

### Ethical consideration and informed consent

This study adhered to the principles of the Helsinki Declaration and the Strengthening the Reporting of Observational Studies in Epidemiology (STROBE) Statement. Ethical principles of autonomy, confidentiality, and anonymity were considered. The ethical review board at the Institute for Endocrine Science, Shahid Beheshti University of Medical Science approved the study with the ethical number of IR.SBMU.ENDOCRINE.REC.1401.069. Medical staff from the research team thoroughly explained the study protocol to each participant individually. Written informed consent was obtained from all participants, and in cases of refusal, the participant was excluded from the study. The informed consent included permission for data collection, performing the required laboratory tests, and publication while maintaining confidentiality principles.

### Tehran lipid and glucose study

This prospective cohort study is based on the Tehran Lipid and Glucose (TLGS) cohort, which is a longitudinal population-based cohort aimed at investigating non-communicable diseases in Tehran, Iran. TLGS study included fifteen thousand adult residents in the eastern area of Tehran, Iran using a multistage cluster random sampling method. Participants were followed every three years, and data was collected from 1999 to 2021. The full details of TLGS were previously described^19,20^.

All participants invited to the TLGS unit are referred to experienced physicians after providing their informed written consent. These physicians conduct interviews to gather participants’ past medical history and complete a comprehensive 110-item questionnaire. This questionnaire covers a range of topics, including family history of NCD, smoking habits, reproductive history, and physical activity assessment. A brief physical examination, including anthropometric measurements, is also performed. Dietary data for one-tenth of the participating families is collected by trained dietitians.

Education levels are categorized into three groups: primary (up to 6 years), secondary (6–12 years), and tertiary education (over 12 years). Physical activity levels are quantified using METS derived from activity questionnaires, with less than 600 min/week indicating low activity. Physicians trained in obtaining anthropometric measurements record WC, weight, and height following standard protocols, and BMI is calculated accordingly. Participants are asked to remain seated for 15 min, after which a qualified physician measures blood pressure twice using a standard mercury sphygmomanometer calibrated by the Iranian Institute of Standards and Industrial Researches.

### Biochemical analysis

Upon admission, personal characteristics are documented, and a unique computer code is assigned. A 10-mL sample of venous blood is collected from all study participants between 7:00 and 9:00 a.m. after a 12–14 h overnight fast. The blood samples are taken in a sitting position following a standard protocol and are kept for one and a half hours under standard lab conditions.

All laboratory kits are provided by Pars Azmon Inc., Iran. Serum total cholesterol and triglycerides (TG) are measured using enzymatic calorimetric tests with cholesterol esterase and cholesterol oxidase, and glycerol phosphate oxidase, respectively. HDL is measured after the precipitation of apolipoprotein B containing lipoproteins with phosphotungistic acid. Assay performance is monitored every 20 tests using the lipid control serum, Precinorm [normal range] and Precipath [pathologic range] (Boehringer Mannheim, Germany; cat. no. 1446070 for Precinorm and 171778 for Precipath).

Serum glucose concentration is assayed using an enzymatic colorimetric method with a glucose oxidase technique to assess fasting blood glucose (FBS). Assay performance is monitored every 20 tests using the glucose control serum, Precinorm [normal range] and Precipath [pathologic range] (Boehringer Mannheim, Germany; cat. no. 1446070 for Precinorm and 171778 for Precipath). A glucose standard (C.f.a.s, Roche, Germany; cat. no. 759350) is used to calibrate the Selectra 2 auto-analyzer on all days of laboratory analyses. All samples are analyzed when internal quality control meets the acceptable criteria. Inter- and intra-assay coefficients of variations are both 2.2% for serum glucose and 0.6% for TGs^19^. Serum creatinine (Cr) is measured according to the standard colorimetric Jaffe-Kinetic reaction method (Pars Azmon Inc, Tehran, Iran).

### Study design and exclusion criteria

In this study, we collected available data for demographic data (age, sex), laboratory exams (FBS, HDL, TG, Cr), smoking status, education, physical activity, and hemodynamic indicators (systolic and diastolic blood pressure) from the accessible TLGS database. Participants completed the sixth round of follow-up every three years, resulting in a minimum of 18 years of prospective observation.

**Figure 1:**
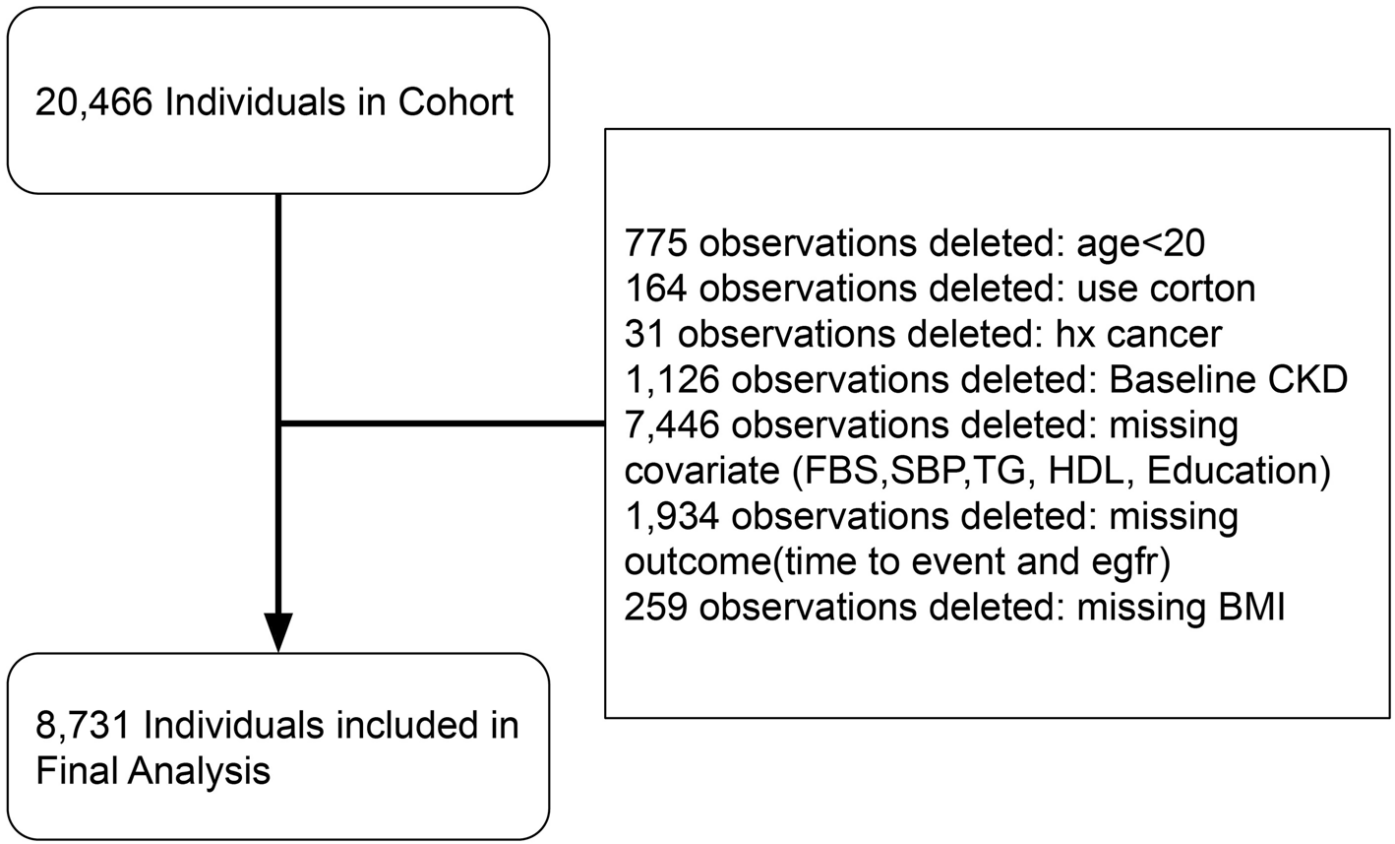
Patient flow diagram of patients and individuals included in the final analysis.

We selected participants for our study from the pool of participants in the TLGS cohort based on the following criteria: availability of valid data in phases of the TLGS cohort, and exclusion of participants who had missing values in covariates or target variable of glomerular filtration rate (GFR), were under 20 years old, diagnosed with cancer during the study period, or received corticosteroid treatments (Fig. 1). From this enrolled cohort, we excluded participants who met any of the following criteria: (1) eGFR < 60 mL/min at baseline; (2) follow-up duration of less than one year; (3) lack of conclusive data on metabolic and renal function.

### Aim and outcome

This research aimed to answer the following questions:

- Primary Objective 1: Evaluate the risk of CKD incidence among four obesity-metabolic phenotypes.
- Primary Objective 2: Determine the impact of participants’ state transitions on CKD risk.
- Extended Investigation 1: Assess the adjusted effect of each component of metabolic health on CKD risk.
- Extended Investigation 2: In the context of defining obesity-metabolic health states, what is the impact of the number of metabolic health components, with or without obesity, on CKD risk?
- Extended Investigation 3: In terms of defining obesity-metabolic health states, what is the concordance between using WC and BMI for defining obesity?

The main outcome of study was the occurrence of CKD, defined as having two GFR < 60 mL/min/1.73m2 in two phases of TLGS cohort which have three years interval. The CKD-EPI equation was used for GFR calculation, which is as follows^21^:

$$GFR=141 \times {{\text{min}}(\frac{Scr}{k}, 1)}^{\alpha } \times {{\text{max}}(\frac{Scr}{k}, 1)}^{-1.209}\times {0.993}^{Age}\times 1.018[if \;female]$$

where Scr is serum creatinine, κ is 0.7 for females and 0.9 for males, α is -0.329 for females and -0.411 for males, min indicates the minimum, and max indicates maximum.

### Definition of metabolically health, obesity, and four phenotypes of their interplay

The definition of metabolic health varies across studies and is subject to debate^15^. In this study, we utilized our previously validated definition of metabolic health, which is based on an evidence-based approach combined with expert opinions using the Delphi method^15^. The consensus among experts to exclude Waist Circumference (WC) from the metabolic health definition aligns with the National Cholesterol Education Program Adult Treatment Panel III^22^. Consequently, the metabolically unhealthy condition in this study was defined as the presence of three or four of the following criteria:

1. Decreased HDL (< 40 mg/dL in men or < 50 mg/dL in women).
2. High TG (≥ 150 mg/dL).
3. High FBS (≥ 100 mg/dL) or use of anti-glycemic oral medication for glycemic control.
4. Systolic blood pressure equal to or above 130 mmHg OR diastolic blood pressure equal to or above 85 mmHg or antihypertensive medication.

Obesity was defined as BMI greater than 30 kg/m^2^. For the purpose of investigating the concordance of obesity-metabolic health and WC-metabolic health, we defined abnormal WC as ≥ 95 cm, according to the cutoff points for Iranian adults of both genders^23^. Based on the state of obesity and the presence of metabolic syndrome components, participants were categorized into four major groups:

- MH-NO Phenotype: This refers to individuals who exhibit a metabolically healthy profile and do not have obesity (Metabolically Healthy, No Obesity: MH-NO).
- MU-NO Phenotype: This category includes individuals who have a metabolically unhealthy profile but do not have obesity (Metabolically Unhealthy, No Obesity: MU-NO).
- MH-O Phenotype: This group consists of individuals who have a metabolically healthy profile but have obesity (Metabolically Healthy, Obesity: MH-O).
- MU-O Phenotype: This phenotype pertains to individuals who have a metabolically unhealthy profile and also have obesity (Metabolically Unhealthy, Obesity: MU-O).

### Statistical analysis

The statistical analysis was performed using Stata (StataCorp. 2015. Stata statistical software: Release 14, College Station, TX: StataCorp LP.). A p-value of less than or equal to 0.05 was considered statistically significant. The baseline characteristics of participants were expressed as mean, standard deviation, median, and interquartile range (IQR) for continuous variables and frequency (%) for ordered variables. The baseline characteristics of participants between four groups were compared by the Student's t-test and Chi-square test for continuous and ordered variables, respectively. We used the Cox proportional hazard test to analyse the association between obesity phenotype and metabolic unhealthy profile with CKD. We defined survival time as the interval between study inclusion and CKD advent or censoring. Also, the event time was defined as half-time survival between the first diagnosis of CKD and the last normal laboratory result. The univariate Cox regression analysis was performed for all confounding factors such as age, sex, BMI, and smoking.

Additional variables with a p-value of less than 0.2 in the univariate study were considered for multivariate regression model analysis. The first statistical model (unadjusted) showed crude rates. The second model (age-sex adjusted) was adjusted for age and sex. The third model (fully adjusted) was adjusted for age and sex (female, male), and the third analysis was adjusted for age, sex, smoking (smoker, non-smoker), education (primary education, college undergrad, postgrad), and physical activity (Metabolic Equivalent of Task < 600, > 600). The Schoenfeld residual test was used to examine the proportional hazard assumption in the Cox model. The cumulative incidence rate of CKD was calculated as new cases divided by the at-risk individual-time during the follow-up period. Additionally, the effect of each component on the total cohort was evaluated after adjusting for covariates and other components.

Finally, three hazard ratios and their corresponding 95% confidence interval (presented as HR [lower confidence interval, upper confidence interval] of CKD incident were calculated using three Cox tests: (1) Unadjusted, (2) Age and sex adjusted, (3) Fully adjusted (called as adj-HR) (age, sex, smoking, education, physical activity). For reports of HRs stratified by gender, the stratified variable was excluded from the adjusted models.

## Results

As illustrated in Fig. 1, out of 20,466 individuals in the TLGS cohorts, 8731 participants were included in the final analysis. The characteristics of the 1934 participants who were excluded due to missing outcomes were comparable to those of the enrolled cohort (Supplementary Table S1), with the exception of education (primary education: 7.19% vs 35.06% in the enrolled cohort). The included participants were divided into four subgroups: MH-NO, MU-NO, MH-O, and MU-O, consisting of 5645, 1163, 1212, and 711 individuals, respectively. The average age of the total cohort was 39.93 ± 13.22 years, and 55.8% of the participants were female. Nearly half of the participants (45.47%) had low physical activity, and less than one-sixth of the participants (13.94%) had a history of smoking. The mean BMI of the total participants was 26.68 ± 4.67 kg/m^2^, and the mean BMI of the MH-NO, MU-NO, MH-O, and MU-O groups were 24.50 ± 3.18 kg/m^2^, 26.50 ± 2.37 kg/m^2^, 32.90 ± 2.84 kg/m^2^, and 33.72 ± 3.35 kg/m^2^, respectively (p < 0.001).

The most common comorbidities in the total cohort were dyslipidemia, hypertension, and diabetes mellitus, present in 70.94%, 27.09%, and 12.36% of participants, respectively. The prevalence of comorbidities was significantly higher in the MU-O group (p < 0.001). It is noteworthy that antihypertensive medications were the most commonly used, followed by anti-diabetic and lipid-lowering medications. Table 1 summarizes the baseline demographic characteristics, comorbidities, medications, and metabolic risk factors of subjects with chronic kidney disease over an 18-year follow-up period.

**Table 1:** Baseline characteristics of subjects in different metabolic syndrome-obesity.

	Total n = 8731	MH-NO n = 5645	MU-NO n = 1163	MH-O n = 1212	MU-O n = 711	p-value
	Mean ± SD or number (%)	Mean ± SD or number (%)	Mean ± SD or number (%)	Mean ± SD or number (%)	Mean ± SD or number (%)	
Age, year	39.93 ± 13.22	37.00 ± 12.84	48.48 ± 12.41	40.69 ± 11.29	47.87 ± 10.84	< 0.001
Female	4809 (55.08)	2962 (52.47)	507 (43.59)	855 (70.54)	485 (68.21)	< 0.001
Primary Education	3061 (35.06)	1539 (27.26)	559 (48.07)	544 (44.88)	419 (58.93)	< 0.001
Ever smokers	1217 (13.94)	878 (15.55)	155 (13.33)	112 (9.24)	72 (10.13)	< 0.001
Low physical activity	3970 (45.47)	2644 (46.84)	521 (44.80)	530 (43.73)	275 (38.68)	< 0.001
Body mass index (kg/m^2^)	26.68 ± 4.67	24.50 ± 3.18	26.50 ± 2.37	32.90 ± 2.84	33.72 ± 3.35	< 0.001
Comorbidity						
DM	1078 (12.36)	256 (4.54)	376 (32.41)	140 (11.56)	306 (43.10)	< 0.001
HTN	2351 (27.09)	670 (11.95)	877 (75.54)	248 (20.67)	556 (78.20)	< 0.001
Dyslipidemia	6185 (70.94)	3570 (63.34)	1103 (94.92)	844 (69.69)	668 (94.08)	< 0.001
Medication						
Antihypertensive	428 (4.92)	98 (1.74)	152 (13.13)	43 (3.57)	135 (19.01)	< 0.001
Anti-diabetic	278 (3.19)	56 (1.00)	115 (9.91)	12 (0.99)	95 (13.36)	< 0.001
Lipid-lowering	223 (2.56)	43 (0.76)	96 (8.28)	16 (1.32)	68 (9.58)	< 0.001
Laboratory data						
eGFR at earliest (mL/min 1.73 m^2^)	76.81 ± 10.67	84.02 ± 12.87	76.93 ± 11.55	79.78 ± 12.16	76.09 ± 10.87	< 0.001
eGFR at last follow-up (mL/min 1.73 m^2^)	71.49 ± 13.34	73.47 ± 12.87	66.20 ± 13.12	70.75 ± 13.40	65.75 ± 13.15	< 0.001
SBP, mmHg	117.22 ± 17.42	111.91 ± 14.08	132.02 ± 18.51	117.64 ± 15.15	134.25 ± 18.03	< 0.001
DBP, mmHg	76.81 ± 10.67	73.48 ± 9.18	84.98 ± 10.31	78.27 ± 9.11	87.31 ± 10.06	< 0.001
WC, cm	87.90 ± 12.19	82.68 ± 9.77	90.69 ± 8.32	99.93 ± 8.79	104.13 ± 9.11	< 0.001
FBG, mg/dL	96.10 ± 29.42	89.15 ± 16.51	117.94 ± 47.57	91.68 ± 17.15	123.19 ± 48.41	< 0.001
TG, mg/dL	166.39 ± 118.65	134.90 ± 90.15	263.17 ± 154.41	164.12 ± 88.59	262.41 ± 154.03	< 0.001
HDL-C, mg/dL	41.51 ± 10.83	42.91 ± 11.05	35.85 ± 7.96	42.58 ± 11.13	37.80 ± 8.56	< 0.001
TC, mg/dL	206.39 ± 45.20	196.56 ± 42.01	225.83 ± 47.44	213.75 ± 40.67	236.51 ± 45.78	< 0.001

*MH-NO* Metabolically Healthy, No Obesity; *MU-NO* Metabolically Abnormal, No Obesity; *MH-O* Metabolically Healthy, Obesity; *MU-O* Metabolically Abnormal, Obesity; *DM* Diabetes Mellitus; *HTN* Hypertension; *eGFR* estimated Glomerular Filtration Rate; *SBP* Systolic Blood Pressure; *DBP* Diastolic Blood Pressure; *WC* Wrist Circumflex; *FBG* Fasting Blood Glucose; *TG* Triglyceride; *HDL-C* High-Density Lipoprotein; *TC* Total Cholesterol.

### Primary Aim 1: CKD risk among four obesity-metabolic health phenotypes

**Figure 2:**
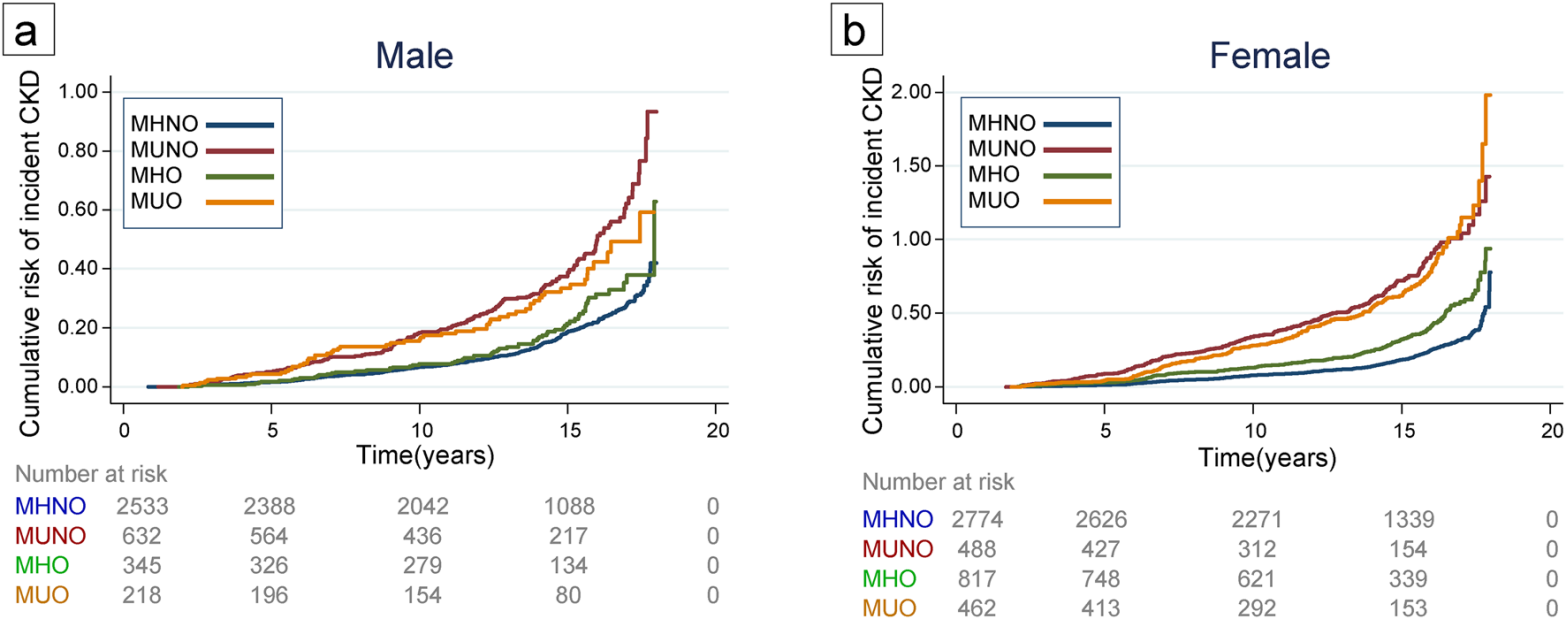
Cumulative risk curve of CKD incident among four phenotypes of *MH-NO* Metabolically Healthy, No Obesity; *MU-NO* Metabolically Unhealthy, No Obesity; *MH-O* Metabolically Healthy, with Obesity; *MU-O* Metabolically Unhealthy with Obesity among (**a**) male and (**b**) female.

Table 2 presents the incidence and risk of CKD progression in the total cohort, as well as in females and males, according to three previously defined models. As indicated, when participants are adjusted for age and sex, both the MU-NO and MU-O subgroups have an equal risk of CKD progression, which is 2.93 times higher compared to the MH-NO cohort. When adjustments are made for smoking status and BMI, the MU-O group exhibits the highest risk of CKD progression (Adj-HR: 1.63 [1.42–1.87]). Figure 2 illustrates the comparable impact of this increased risk of CKD between males and females.

**Table 2:** Incidence rates and risk of the CKD in metabolic syndrome-obesity phenotype status.

	Person-years	CKD Case	Incidence rate (per 1000 PYs)	Crude model^a^	Age-sex adjusted model^b^	Fully adjusted model^c^
				HR (95% CI)	HR (95% CI)	HR (95% CI)
Male						
MH-NO	35,838	409	11.41	1 (Reference)	1 (Reference)	1 (Reference)
MU-NO	7990	201	25.15	2.38 (2.01–2.82)	1.62 (1.37–1.92)	1.63 (1.37–1.93)
MH-O	4684	65	13.87	1.26 (0.97–1.64)	1.25 (0.96–1.62)	1.30 (1.00–1.69)
MU-O	2764	59	21.33	2.02 (1.53–2.65)	1.54 (1.17–2.02)	1.58 (1.20–2.08)
Female						
MH-NO	40,368	536	13.27	1 (Reference)	1 (Reference)	1 (Reference)
MU-NO	5929	257	43.34	3.70 (3.19–4.30)	1.32 (1.13–1.54)	1.33 (1.13–1.55)
MH-O	11,018	237	21.51	1.70 (1.46–1.98)	1.12 (0.96–1.31)	1.14 (0.98–1.33)
MU-O	5714	220	38.49	3.25 (2.78–3.80)	1.30 (1.11–1.53)	1.33 (1.13–1.56)
Total						
MH-NO	76,207	945	12.40	1 (Reference)	1 (Reference)	1 (Reference)
MU-NO	13,919	458	32.90	2.93 (2.62–3.28)	1.51 (1.35–1.69)	1.49 (1.33–1.67)
MH-O	15,702	302	19.23	1.62 (1.42–1.84)	1.37 (1.20–1.56)	1.35 (1.18–1.54)
MU-O	8479	279	32.90	2.93 (2.56–3.35)	1.65 (1.44–1.89)	1.63 (1.42–1.87)

^a^Model 1: crude rate (no adjustment); ^b^Model 2: adjusted for age and sex; ^c^Model 3: adjusted for age, sex, smoking, education, and physical activity. *CKD* Chronic Kidney Disease; *PY* Person-Year; *HR* Hazard Ratio; *MH-NO* Metabolically Healthy, No Obesity; *MU-NO* Metabolically Unhealthy, No Obesity; *MH-O* Metabolically Healthy, Obesity; *MU-O* Metabolically Unhealthy, Obesity.

The extended analysis of overweight and metabolic health, as shown in Supplementary Table S2, indicates that being overweight can independently increase the risk of CKD, even in metabolically healthy individuals (Adj-HR: 1.20 [1.05–1.36]). However, the impact of being metabolically unhealthy in the normal weight group (Adj-HR: 1.60 [1.29–1.99]) surpasses that of being overweight but metabolically healthy.

We also examined the risk of CKD progression among participants younger and older than 50, as detailed in Table 3. It’s noteworthy that in both age groups, the MU-NO cohort has a higher risk of CKD progression compared to the MH-NO cohort (Younger-than-50 HR: 1.42 [1.15–1.75], and Older-than-50 HR: 1.36 [1.18–1.58]).

**Table 3:** Incidence rates and risk of the CKD disease in obesity-metabolic phenotype status by age, 20 years follow up.

	Total no.	CKD cases no. (%)	< 50 years, n = 2862		≥ 50 years, n = 3877		p value interaction
			Incidence rate*	HR (95% CI)	Incidence rate*	HR (95% CI)	
Male	3922	734 (18.7)					
MH-NO	2683	409 (15.2)	4.94	1 (Reference)	9.57	1 (Reference)	–
MU-NO	656	201 (30.6)	12.59	2.65 (1.98–3.56)	25.35	2.50 (1.97–3.17)	< 0.001
MH-O	357	65 (18.2)	7.77	1.68 (1.13–2.51)	15.12	1.54 (1.26–1.87)	0.019
MU-O	226	59 (26.1)	7.19	1.54 (0.87–2.72)	24.04	2.32 (1.82–2.96)	0.60
Female	4809	1250 (26.0)					
MH-NO	2962	536 (18.1)	39.58	1 (Reference)	54.70	1 (Reference)	–
MU-NO	507	257 (50.7)	48.07	1.31 (1.06–1.61)	69.44	1.42 (1.15–1.75)	< 0.001
MH-O	855	237 (27.7)	37.82	0.90 (0.64–1.29)	53.56	1.00 (0.78–1.29)	0.005
MU-O	485	220 (45.3)	47.98	1.29 (0.94–1.77)	61.93	1.24 (1.0–1.56)	< 0.001
Total	8731	1984 (22.7)					
MH-NO	5645	945 (16.7)	7.53	1 (Reference)	44.58	1 (Reference)	–
MU-NO	1163	458 (39.4)	17.75	1.42 (1.15–1.75)	57.92	1.36 (1.18–1.58)	< 0.001
MH-O	1,212	302 (24.9)	13.00	1.00 (0.78–1.29)	48.17	0.97 (0.79–1.18)	< 0.001
MU-O	711	279 (39.2)	18.34	1.24 (0.99–1.56)	57.67	1.24 (1.04–1.49)	< 0.001

*CKD incidence rate is reported per 1000 Person-Years. Cox model was adjusted for age, sex, smoking, education, and physical activity. *CKD* Chronic Kidney Disease; *HR* Hazard Ratio; *MH-NO* Metabolically Healthy, No Obesity; *MU-NO* Metabolically Unhealthy, No Obesity; *MH-O* Metabolically Healthy, with Obesity; *MU-O* Metabolically Unhealthy, Obesity.

### Primary Aim 2: transition of participants’ state and risk of CKD

**Figure 3:**
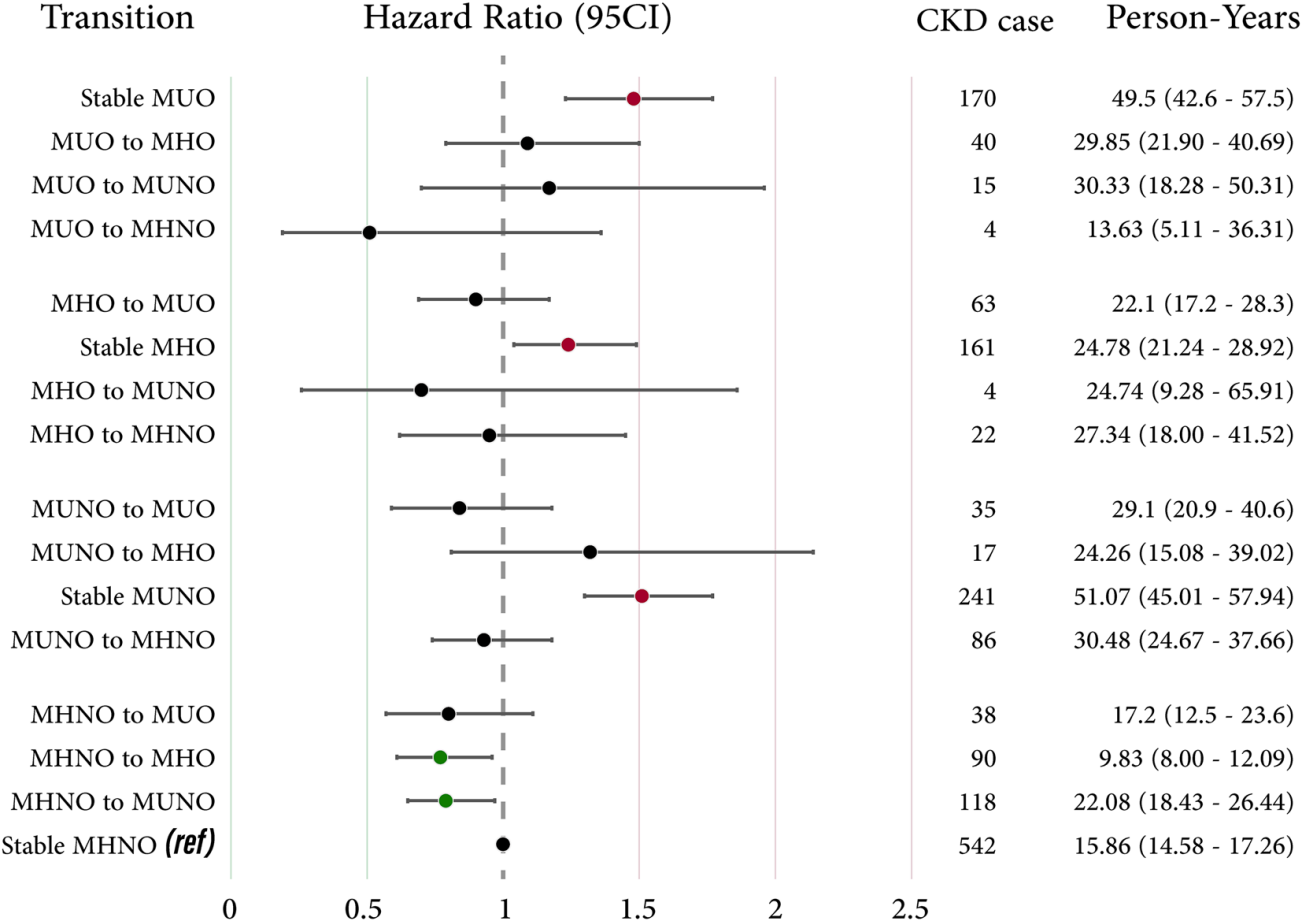
Age-sex adjusted risk of chronic kidney disease according to the transition of Obesity metabolic phenotype in twenty years follow-up. *MH-NO* Metabolically Healthy, No Obesity; *MU-NO* Metabolically Unhealthy, No Obesity; *MH-O* Metabolically Healthy, with Obesity; *MU-O* Metabolically Unhealthy with Obesity, *95 CI* 95% confidence interval.

**Figure 4:**
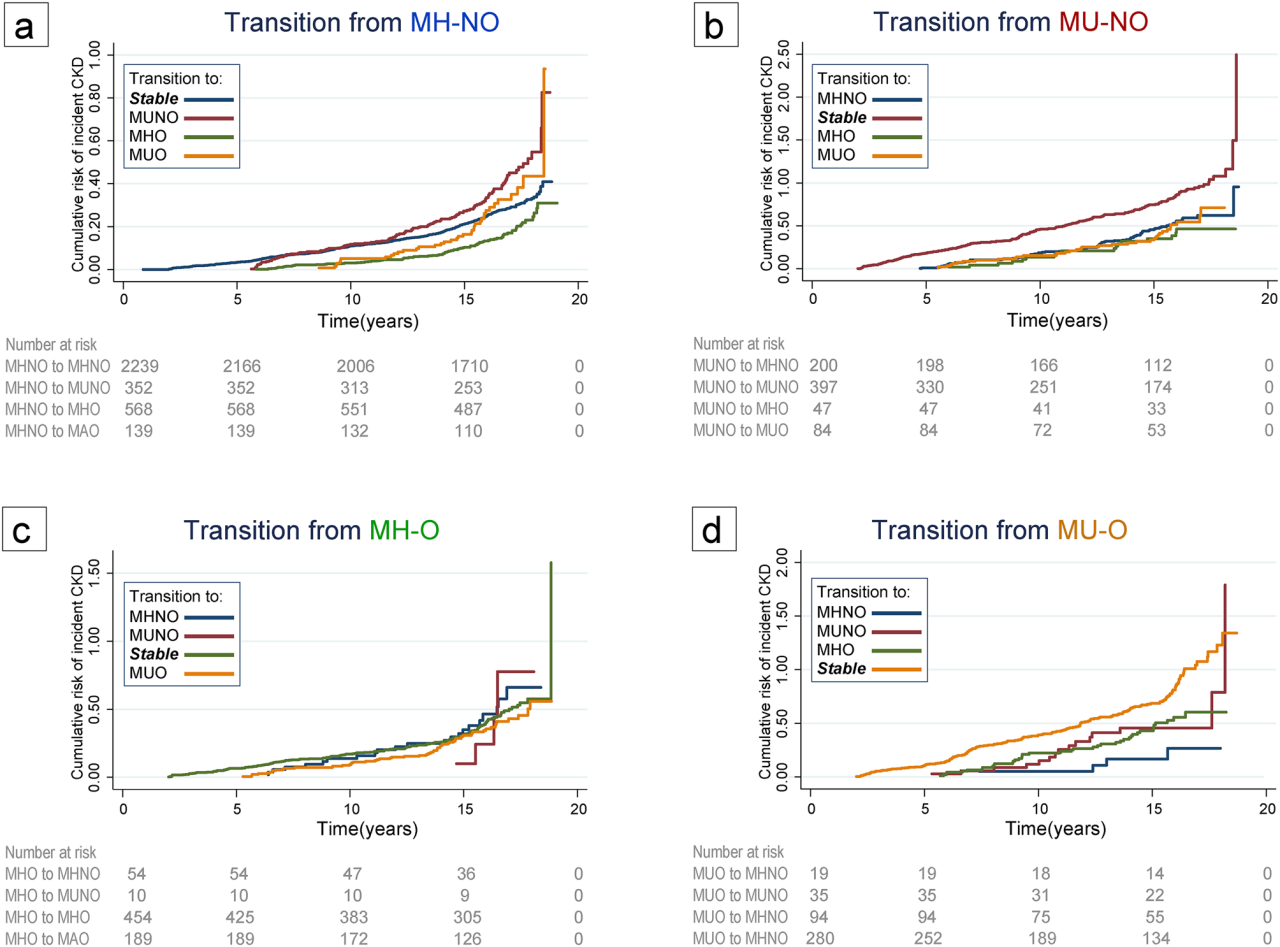
Cumulative risk curve of CKD incident state transitions among four phenotypes of: (**a**) MH-NO: Metabolically Healthy, No Obesity; (**b**) MU-NO: Metabolically Unhealthy, No Obesity; (**c**) MH-O: Metabolically Healthy, with Obesity; (**d**) MU-O: Metabolically Unhealthy with Obesity.

Figure 3 (and Supplementary Table S3) illustrates the dynamics of each cohort of individuals, their phenotypic subgroup changes at the end of the follow-up period, and the subsequent HR of CKD progression in each phenotype alteration. Persistence of MU-NO and MU-O states were associated with the highest risk of CKD progression (HR: 1.51 [1.30–1.77] and HR: 1.48 [1.23–1.77], respectively). Notably, changing the phenotype from MH-NO to either MH-O or MU-NO reduces the risk of CKD progression (HR: 0.77 [0.61–0.96] and HR: 0.79 [0.65–0.97], respectively). Figure 4 displays the cumulative risk of state transitions among phenotypes.

### Extended investigation 1: the adjusted effect of each metabolic health component on CKD

After adjusting for the impact of other components, we investigated the impact of each metabolic health phenotype (TG, BP, FBS, and HDL) on CKD incidence among each phenotype group (Supplementary Table S4) and the total cohort (Supplementary Table S5). High BP poses the highest risk to CKD (HR 1.28 [1.17–1.41]), followed by Obesity (HR 1.20 [1.09–1.33]), high TG (HR 1.28 [1.03–1.25]) and high FBS (HR 1.16 [1.05–1.28]). Interestingly, low HDL alone cannot increase the risk of CKD (P = 0.128).

### Extended investigation 2: the number of MU components and CKD Risk

Supplementary Table S6 describes the risk of CKD in individuals with 0, 1, 2, 3, or 4 abnormal metabolic indices—metabolic health components. The adj-HR becomes statistically significant when the number of abnormal indices reaches 3 components in patients without obesity (Adj-HR 1.48 [1.23–1.78]; reference: 0 components without obesity), while in the obesity group any number of components failed to become statistically significant (reference: 0 components with obesity). However, the unadjusted HR becomes statistically significant when the number of components is 2 in the non-obesity group (unadjusted HR 1.76 [1.48–2.10]) and 3 in the obesity group (unadjusted HR 1.91 [1.24–2.95]). This may suggest that classifying patients with 3 abnormal metabolic indices may be an optimal cut-off point for classifying the metabolically unhealthy group.

### Extended investigation 3: concordance of BMI and WC findings

To investigate the concordance of using WC as an abdominal obesity index and BMI as a general obesity measure, we replicated our results using abnormal WC instead of abnormal BMI, i.e., obesity (Supplementary Table S7). The adjusted model showed the highest risk of CKD incidence is among the metabolically unhealthy-normal WC group (adj-HR 1.68 [1.44–1.95]), followed by the metabolically unhealthy-abnormal WC group (adj-HR 1.43 [1.27–1.61]). Interestingly, the metabolically healthy-abnormal WC group does not show an increased risk of CKD, in either the age-sex adjusted or fully adjusted model. This investigation shows the concordance of BMI and WC, indicating a more prominent role of metabolic health in CKD development. Additionally, being metabolically healthy and having abnormal BMI imposes a greater risk of CKD, compared to being metabolically healthy and having abnormal WC in both the adjusted (adj-HR 1.35 [1.18–1.54] vs 1.09 [0.97–1.22]) and unadjusted model (HR 1.62 [1.42–1.84] vs 1.75 [1.56–1.95]).

## Discussion

**Figure 5:**
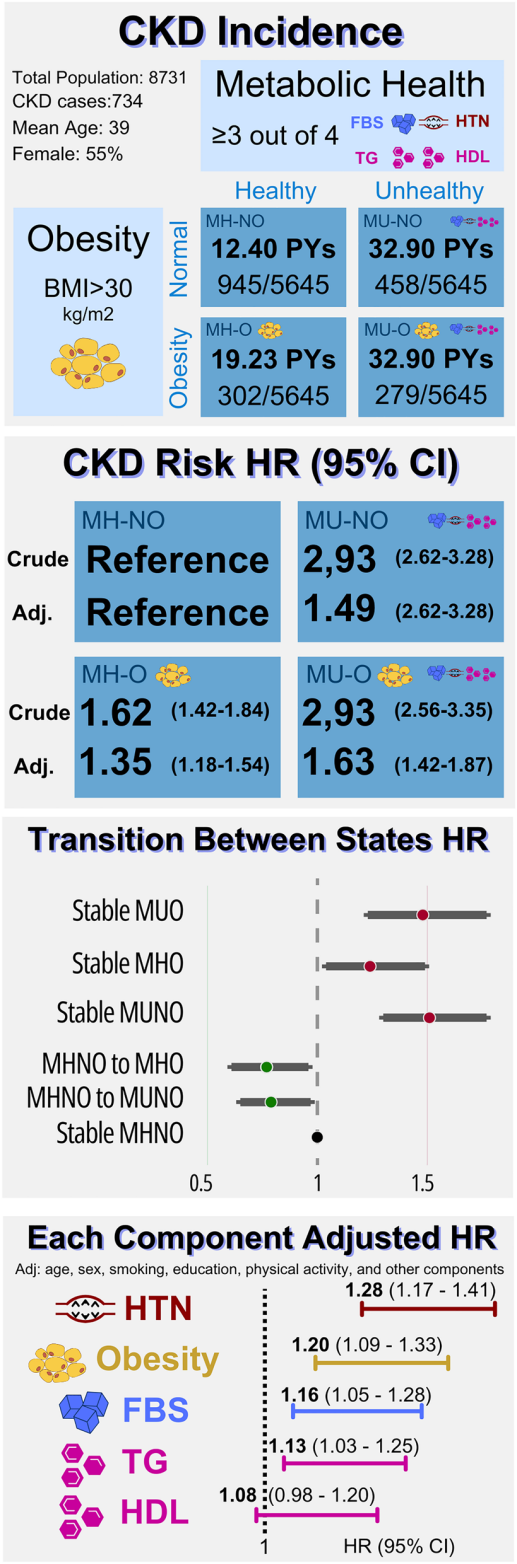
The summary of findings on chronic kidney diseases (CKD) risk among four phenotypes of Metabolically Healthy-No Obesity (MH-NO), Metabolically Unhealthy-No Obesity (MU-NO), Metabolically Healthy-obesity (MH-O), and Metabolically Unhealthy-obesity (MU-O). The full detail was reported in Tables 2, 3, Figs. 2, 3, and Supplementary Table S5. The adjusted HR were reported after adjusting for age, sex, education, smoking, and physical activity for CKD risk HR. The age-sex adjusted model was used for finding transition between states HR, and statistically significant HRs were presented.

Figure 5 offers a concise summary of our prospective cohort study's findings. The investigation highlighted that individual in the MU-O group faced the highest risk of CKD, with an increased risk of 42–87%. This risk was closely followed by the Metabolically MU-NO and MH-O groups, showing elevated risks of 33–67% and 18–54%, respectively. These findings suggest a more substantial influence of metabolic health on CKD progression compared to obesity alone. Our exploration into phenotype state transitions revealed surprising outcomes, particularly the apparent reduction in CKD incidence when transitioning from Metabolically Healthy-No Obesity MH-NO to MHO. In the forthcoming sections, we will discuss our findings regarding exposure time to obesity, ambiguities in phenotype transition, the role of metabolic health components, the underlying pathophysiology, and the importance of a biopsychosocial approach for interventions.

A key observation from our 20-year follow-up study was the vulnerability of MH-O individuals to CKD, contrasting with findings from our previous 10-year follow-up, which negated this impact in the MH-O group^24^. This discovery emphasizes the importance of obesity exposure duration, a concept previously posited in various conditions, including DM, CVD, and cancer^5,25–27^. Notably, in our participants under 50 years, the hazard of CKD was high for MU-NO but not for MH-O, suggesting a more immediate impact of metabolic unhealthiness on CKD incidence compared to the longer-term effects of obesity.

This variance in risk may elucidate why certain cohorts with shorter follow-up periods did not identify obesity without metabolic health issues as a significant CKD risk factor^24,28^. This observation aligns with the "friendly fat theory," which posits that in its early stages, obesity may not be detrimental due to adipocytes' ability to store lipids. However, when this storage capacity is exceeded, the accumulation of fat in ectopic organs such as the liver, visceral tissues, and pancreas can trigger the pathological processes affecting other organs^5,29^. This may also elucidate our unexpected finding that transitioning from MH-NO to MHO could reduce CKD incident (Fig. 3), highlighting the necessity for long-term studies to understand obesity's evolving health impacts fully.

Other studies have put forward an alternative perspective, suggesting that the MH-O state is dynamic, often transitioning to the MU-O phenotype over six to ten years and may evolve into the MU-O phenotype^5,13,30,31^. This transitional nature of the MH-O phenotype is well-documented, yet few studies have explored these dynamics in depth, focusing instead on prevalence rates^18,32^. A meta-analysis encompassing six studies indicated that this transitional state augments cardiovascular risk, despite some heterogeneity in the findings^33^. Our research on state transitions, as illustrated in Figs. 3 and 4, did not identify increased risk with transitions to more adverse phenotypes, diverging from previous CKD-focused cohort studies^18,32^. Our smaller sample size and extended interval between baseline and final phenotype assessments may have influenced these results. Additionally, our definition of metabolic health, based on two or fewer abnormal indices, differs from other studies that categorize such patients as metabolically unhealthy^18^.

Our findings highlight the importance of the persistence of certain states, particularly the MU-NO and MU-O states, in CKD progression, suggesting that initial metabolic status is more critical in determining CKD risk than phenotype transitions. This emphasizes the potential importance of early-life exposure to unhealthy metabolic states and suggests that the impacts of transitioning to more unfavorable phenotypes might emerge in longer-term studies. Thus, extended follow-up could reveal the delayed effects of worsening metabolic phenotypes on CKD risk.

Our insights into CKD incidence among the four obesity phenotypes contribute to resolving controversies, particularly within the MU-O subgroup, as corroborated by a prior meta-analysis of nine cohorts^17^. However, variations exist among studies in terms of follow-up duration, definitions of metabolic unhealthiness, and obesity metrics. We corroborated our approach with an extended analysis showing the concordance of WC and BMI, with BMI demonstrating a more pronounced impact on CKD. Aligned with previous research, we identified being overweight as a precursor to CKD, underscoring the importance of early intervention^17^. We established that three abnormal indices constitute the optimal threshold for identifying at-risk patients, in line with our scoping review and panel discussion outcomes^15,22^.

Defining metabolic health as having two or fewer components of high blood pressure, FBS, TG, and low HDL, our study adjusted for variables like age, education, physical activity, and smoking, revealing hypertension as the highest CKD risk factor (28%), followed by obesity (20%), impaired glucose profile (16%), and high TG (13%). However, HDL disturbance did not demonstrate a similar risk. The 63% increase in CKD risk in the MU-O phenotype calls for future studies to consider these diseases collectively. Supplementary Figure S1 illustrates the molecular pathways leading to CKD involving interconnected pathophysiology and risk factors, including obesity and metabolic components^3,34–36^.

A multitude of biological, psychological, and social factors can contribute to the progression of these diseases^37^. All three aspects should be considered in efforts to minimize the risk of obesity, MetS, and CKD. Specifically, treatments and surgeries can address the biological aspect, early education and healthy behavior can address the psychological aspect, and policymaking and social support should be considered at the social level. It is also evident that each of these five diseases can contribute to the development of one another^3,4^. Given that lifestyle modification is the mainstream of disease prevention and even treatment, physicians may benefit from providing lifestyle interventions and treating each disease individually while assessing the presence of others^2^.

However, several limitations should be considered when interpreting our results. Firstly, our study was limited to participants from the eastern area of Tehran, which may limit the generalizability of our findings to other populations. Additionally, we did not account for the impacts of various other potential confounding factors, including social factors, psychological comorbidities, alcohol consumption, and notably, dietary factors. Our study also defines metabolic health as having 0–2 comorbidities, which may have implications for interpretation since our definition, based on our extended analysis and previous scoping review, may differ from previous studies, resulting in cases with sever dysfunction in metabolic process. Furthermore, we did not employ proteinuria criteria for identifying CKD cases, and the use of three-year intervals for CKD identification may result in some false positive cases among participants. Additionally, our criteria did not include WC in interpreting our results. Therefore, we recommend that future studies be designed as multicenter investigations, accounting for a broader range of confounding factors and utilizing comprehensive criteria for defining metabolic health. Furthermore, we were unable to provide a comprehensive explanation for our findings regarding the transition of states.

## Conclusion

In summary, our study unveils critical insights into the multifaceted nature of CKD risk factors. We discerned that both excess-weight, encompassing obesity and overweight, and metabolic health, alongside its individual components, independently contribute to an increased risk of CKD. Notably, metabolic health exhibits a more pronounced impact. Our findings reveal a temporal distinction in these risk factors' influence on CKD progression: obesity predominantly affects CKD development over longer durations, whereas abnormalities in metabolic health can precipitate CKD more rapidly. These results underscore the pivotal role of early-life metabolic phenotypes in CKD prevention, highlighting a window of opportunity where preventive interventions can have the most profound impact.

To further elucidate the nuances of this complex relationship, comprehensive investigations are essential. These should aim to unravel the intricate pathophysiology underlying the transition between obesity and metabolic health states in CKD progression. A particular focus is warranted on our intriguing observation of a decreased CKD risk and incidence in patients transitioning from a MH-NO state to a MH-O state. This finding, which challenges conventional understanding, invites further exploration into the 'friendly fat theory' and its implications in the realm of CKD. Such in-depth research will not only deepen our understanding of these complex interrelations but also pave the way for more effective, targeted interventions in CKD prevention and management.

### Supplementary Information

Supplementary Information.

## Data Availability

Raw data cannot be shared publicly because of access limit by the research authority. Data are available from the Research Institute for Endocrine Sciences, Shahid Beheshti University of Medical Sciences (contact via Intl_office@sbmu.ac.ir) for researchers who meet the criteria for access to confidential data. Results of analysis are available within manuscript or supplementary material; and further data can be accessible on a reasonable request from corresponding author.

## Abbreviations

CKD: Chronic kidney disease
CVD: Cardiovascular diseases
DM: Diabetes mellitus
MetS: Metabolic syndrome
BMI: Body Mass Index
MH-O: Metabolically healthy obesity
MH-NO: Metabolically healthy-no obesity
MU-NO: Metabolically unhealthy-no obesity
MU-O: Metabolically unhealthy obesity
TLGS: Tehran lipid and glucose study
GFR: Glomerular filtration rate
BP: Blood pressure
FBS: Fasting blood sugar
TG: Triglyceride
HDL: High-density lipoprotein
