# Supplementary material for: Metabolic health's central role in chronic kidney disease progression: a 20-year study of obesity-metabolic phenotype transitions
Source: Sci Rep. 2024 Mar 4;14:5244. doi: 10.1038/s41598-024-56061-x (PMC10912755; doi:10.1038/s41598-024-56061-x)
Supplement: Supplementary file 1 — Supplementary Information. [file 41598_2024_56061_MOESM1_ESM.pdf]

This is a supplementary file to " **Metabolic Health's Central Role in Chronic Kidney Disease Progression: A 20-Year Study of Obesity-Metabolic Phenotype Transitions**" by

Shayesteh Khalili, Seyed Amir Ahmad Safavi-Naini, Paniz Zarand, Safdar Masoumi, Yeganeh Farsi, Farhad Hosseinpanah, Fereidoun Azizi (Corresponding to: Farhad Hosseinpanah; fhospanah@endocrine.ac.ir).

List of Supplementary Material

Supplementary Figure S1. The pathophysiology of metabolic syndrome, insulin resistance, obesity, dyslipidemia, and hypertension resulting in kidney injury and chronic kidney disease (CKD) (1-4). The figure shows how various factors associated with metabolic syndrome can lead to kidney damage and CKD. Hazard ratios used in this figure are adjusted for age, sex, physical activity, education, smoking, and other factors affecting CKD, as presented in Supplementary Table S5. Abbreviations used in the figure are as follows: ROS: Reactive oxygen species; NO: Nitric oxide; LV: Left ventricle; RAS: Renin-angiotensin system; IGF: Insulin-like growth factor; TGF-B: Transforming growth factor-beta; ET-1: Endothelin-1; IGF-1: Insulin-like growth factor 1; TNF: Tumor necrosis factor; IL-6: Interleukin-6; GFR: Glomerular filtration rate; NEFA: Non-esterified fatty acids; SREBP-1: Sterol regulatory element-binding protein-1; LDL: Low-density lipoprotein; HDL: High-density lipoprotein; TG: Triglycerides.

Supplementary Table S1: Descriptive Comparison Between Enrolled and Excluded Cohorts.

Supplementary Table S2. Risk of chronic kidney disease among phenotypes of metabolic health and excess body weight (overweight ant obesity).

Supplementary Table S3: Incidence Rates and Risk of Chronic Kidney Disease (CKD) According to the Transition of Obese Metabolic Phenotypes Over a 20-Year Follow-Up

Supplementary Table S4: Hazard Ratios and 95% Confidence Intervals for Chronic Kidney Disease Incidence Across Metabolic Health Components, Stratified by Metabolic State Subgroups in the 20-Year Follow-Up of the Tehran Lipid and Glucose Study

Supplementary Table S5: Hazard Ratios (HR) for Chronic Kidney Disease Incidence Considering Metabolic Health Components and Covariates in the 20-Year Follow-Up of the Tehran Lipid and Glucose Study

Supplementary Table S6: Impact of Obesity-Metabolic Health on Chronic Kidney Disease (CKD), Stratified by Number of Metabolic Health Components

Supplementary Table S7: Concordance of Waist Circumference (WC) and Obesity (Defined by Body Mass Index) with Chronic Kidney Disease (CKD) in the Context of Metabolic Health

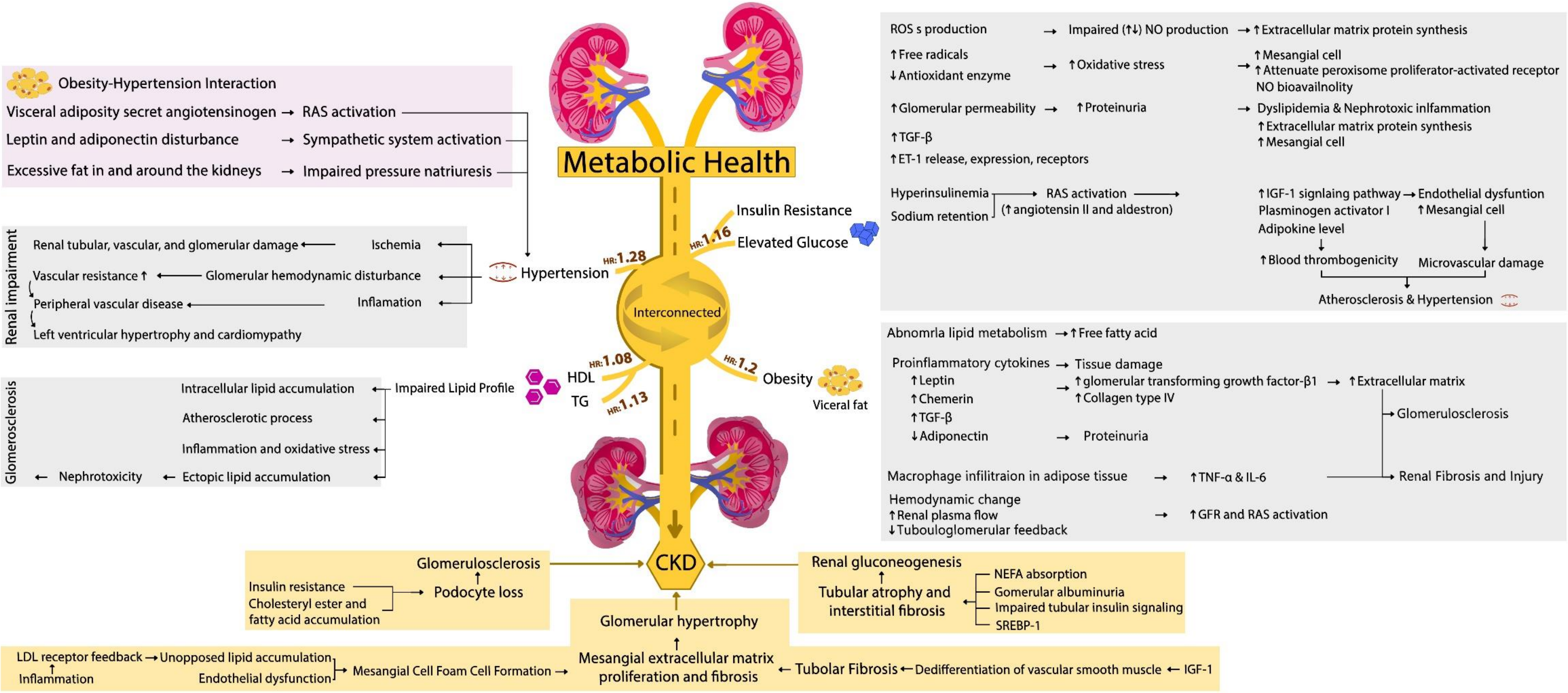

Supplementary Figure S1. The pathophysiology of metabolic syndrome, insulin resistance, obesity, dyslipidemia, and hypertension resulting in kidney injury and chronic kidney disease (CKD) (1-4). The figure shows how various factors associated with metabolic syndrome can lead to kidney damage and CKD. Hazard ratios used in this figure are adjusted for age, sex, physical activity, education, smoking, and other factors affecting CKD, as presented in Supplementary Table S5. Abbreviations used in the figure are as follows: ROS: Reactive oxygen species; NO: Nitric oxide; LV: Left ventricle; RAS: Renin-angiotensin system; IGF: Insulin-like growth factor; TGF-B: Transforming growth factor-beta; ET-1: Endothelin-1; IGF-1: Insulin-like growth factor 1; TNF: Tumor necrosis factor; IL-6: Interleukin-6; GFR: Glomerular filtration rate; NEFA: Non-esterified fatty acids; SREBP-1: Sterol regulatory element-binding protein-1; LDL: Low-density lipoprotein; HDL: High-density lipoprotein; TG: Triglycerides.

Supplementary Table S1: Descriptive Comparison Between Enrolled and Excluded Cohorts.

	Not missing n=8731	Missing Outcome n=1934
	Mean ± SD or Number (%)	Mean ± SD or Number (%)
Age, year	39.93±13.22	40.01 ±15.13
Female	4809 (55.08)	1099 (56.83)
Primary Education	3061 (35.06)	139 (7.19)
Ever smokers	1217 (13.94)	335 (17.32)
Low physical activity	3970 (45.47)	822 (42.50)
Body mass index (kg/m^2)	26.68 ± 4.67	26.63 ± 5.22
Comorbidity		
DM	1078(12.36)	145 (7.52)
HTN	2351(27.09)	562 (29.06)
Dyslipidaemia	6185(70.94)	1357 (70.17)
Medication		
Antihypertensive	428(4.92)	129 (6.79)
Anti-diabetic	278(3.19)	93 (4.90)
Lipid-lowering	223(2.56)	64 (3.37)
Laboratory data		
eGFR (mL/min 1.73 m^2)	76.81±10.67	76.90±10.77
SBP, mmHg	117.22±17.42	118.59±19.59
DBP, mmHg	76.81±10.67	76.91±11.05
WC, cm	87.90±12.19	87.51±12.82
FBG, mg/dl	96.10±29.42	97.92±33.91
TG, mg/dl	166.39±118.65	162.71±114.57
HDL-C, mg/dl	41.51±10.83	41.81±11.16
TC, mg/dl	206.39±45.20	204.98±45.79

Footnote: DM: Diabetes Mellitus; HTN: Hypertension; eGFR: estimated Glomerular Filtration Rate; SBP: Systolic Blood Pressure; DBP: Diastolic Blood Pressure; WC: Wrist Circumflex; FBG: Fasting Blood Glucose; TG: Triglyceride; HDL-C: High-Density Lipoprotein; TC: Total Cholesterol.

Supplementary Table S2. Risk of chronic kidney disease among phenotypes of metabolic health and excess body weight (overweight ant obesity).

	person-years	CKD Case	Incidence rate (Per 1000 PYs)	Crude Model	Age-Sex Adjusted Model	Fully adjusted Model a
				HR (95% CI)	HR (95% CI)	HR (95% CI)
**Male**						
MH-N	19866.568	224	11.3(9.9-12.9)	1 (Reference)	1 (Reference)	1 (Reference)
MU-N	2043.4908	49	24.0(18.1-31.7)	2.35(1.72-3.20)	1.63(1.19-2.22)	1.61(1.18-2.20)
MH-OW	15972.06	185	11.6(10.0-13.4)	1.04(0.85-1.26)	0.96(0.79-1.17)	0.97(0.80-1.18)
MU-OW	5946.6639	152	25.6(21.8-30.0)	2.44(1.99-3.00)	1.58(1.29-1.95)	1.60(1.30-1.97)
MH-O	4684.0055	65	13.9(10.9-17.7)	1.28(0.97-1.69)	1.22(0.93-1.61)	1.27(0.96-1.67)
MU-O	2764.8597	59	21.3(16.5-27.5)	2.05(1.54-2.73)	1.51(1.13-2.01)	1.54(1.16-2.06)
**Female**						
MH-N	20543.737	189	9.2(8.0-10.6)	1 (Reference)	1 (Reference)	1 (Reference)
MU-N	1302.8693	54	41.4(31.7-54.1)	5.09(3.76-6.89)	1.51(1.11-2.05)	1.50(1.10-2.04)
MH-OW	19434.182	343	17.6(15.9-19.6)	2.00(1.67-2.38)	1.28(1.07-1.53)	1.29(1.08-1.54)
MU-OW	4611.2772	202	43.8(38.2-50.3)	5.53(4.54-6.75)	1.56(1.26-1.92)	1.59(1.29-1.96)
MH-O	10985.303	237	21.6(19.0-24.5)	2.51(2.07-3.03)	1.31(1.08-1.59)	1.35(1.11-1.64)
MU-O	5691.0335	220	38.7(33.9-44.1)	4.80(3.95-5.83)	1.53(1.25-1.87)	1.58(1.29-1.93)
**Total**						
MH-N	40410.305	413	10.2(9.3-11.3)	1 (Reference)	1 (Reference)	1 (Reference)
MU-N	3346.36	103	30.8(25.4-37.3)	3.38(2.72-4.19)	1.65(1.33-2.05)	1.60(1.29-1.99)
MH-OW	35406.242	528	14.9(13.7-16.2)	1.50(1.31-1.70)	1.22(1.07-1.38)	1.20(1.05-1.36)
MU-OW	10557.941	354	33.5(30.2-37.2)	3.66(3.18-4.22)	1.69(1.46-1.95)	1.65(1.43-1.91)
MH-O	15669.309	302	19.3(17.2-21.6)	1.99(1.71-2.30)	1.53(1.32-1.77)	1.49(1.28-1.73)
MU-O	8455.8932	279	33.0(29.3-37.1)	3.61(3.10-4.20)	1.84(1.58-2.14)	1.80(1.54-2.10)

Footnote: MH-N: Metabolically Healthy, Normal weight; MU-N: Metabolically Abnormal, Normal weight; MH-OW: Metabolically Healthy, Over Weight; MU-OW: Metabolically Abnormal, Over Weight; MH-O: Metabolically Healthy, Obesity; MU-O: Metabolically Abnormal, Obesity; Model 1: crude rate (no adjustment); a Model 2: adjusted for age and sex; Model 3: adjusted for age, sex, smoking, education, and physical activity.

Supplementary Table S3: Incidence Rates and Risk of Chronic Kidney Disease (CKD) According to the Transition of Obese Metabolic Phenotypes Over a 20-Year Follow-Up

	person-years	CKD Case	Incidence rate Per 1000 PYs	HR (95% CI)
MH-NO → MH-NO	34167	542	15.86 (14.58 - 17.26)	1 (Reference)
MH-NO → MU-NO	5345	118	22.08 (18.43 - 26.44)	0.79 (0.65 - 0.97)
MH-NO → MH-O	9155	90	9.83 (8.00 - 12.09)	0.77 (0.61 - 0.96)
MH-NO → MU-O	2209	38	17.2 (12.5 - 23.6)	0.80 (0.57 - 1.11)
MU-NO → MH-NO	2821	86	30.48 (24.67 - 37.66)	0.93 (0.74 - 1.18)
MU-NO → MU-NO	4719	241	51.07 (45.01 - 57.94)	1.51 (1.30 - 1.77)
MU-NO → MH-O	700	17	24.26 (15.08 - 39.02)	1.32 (0.81 - 2.14)
MU-NO → MU-O	1200	35	29.1 (20.9 - 40.6)	0.84 (0.59 - 1.18)
MH-O → MH-NO	804	22	27.34 (18.00 - 41.52)	0.95 (0.62 - 1.45)
MH-O → MU-NO	161	4	24.74 (9.28 - 65.91)	0.70 (0.26 - 1.86)
MH-O → MH-O	6496	161	24.78 (21.24 - 28.92)	1.24 (1.04 - 1.49)
MH-O → MU-O	2853	63	22.1 (17.2 - 28.3)	0.90 (0.69 - 1.17)
MU-O → MH-NO	293	4	13.63 (5.11 - 36.31)	0.51 (0.19 - 1.36)
MU-O → MU-NO	494	15	30.33 (18.28 - 50.31)	1.17 (0.70 - 1.96)
MU-O → MH-O	1340	40	29.85 (21.90 - 40.69)	1.09 (0.79 - 1.50)
MU-O → MU-O	3435	170	49.5 (42.6 - 57.5)	1.48 (1.23 - 1.77)

Footnote: CKD: Chronic Kidney Disease; PY: Person- Year; HR: Hazard Ratio; MH-NO: Metabolically Healthy, Non-Obese; MU-NO: Metabolically Abnormal, Non-Obese; MH-O: Metabolically Healthy, Obese; MU-O: Metabolically Abnormal, Obese

Supplementary Table S4: Hazard Ratios and 95% Confidence Intervals for Chronic Kidney Disease Incidence Across Metabolic Health Components, Stratified by Metabolic State Subgroups in the 20-Year Follow-Up of the Tehran Lipid and Glucose Study

	MH-NO	MU-NO	MH-O	MU-O
Male*	HR (95% CI)	HR (95% CI)	HR (95% CI)	HR (95% CI)
High TG	1.02(0.83-1.25)	1.19(0.61-2.34)	1.42(0.83-2.43)	1.23(0.30-5.08)
High BP	1.24(0.99-1.56)	**1.52(1.06-2.17)**	1.17(0.67-2.03)	1.45(0.63-3.33)
High FBS	1.04(0.78-1.38)	0.95(0.71-1.27)	**2.07(1.16-3.69)**	1.14(0.60-2.16)
Low HDL	1.01(0.83-1.23)	0.71(0.45-1.12)	0.44(0.26-0.73)	0.60(0.25-1.44)
Female*				
High TG	1.14(0.94-1.37)	0.90(0.57-1.42)	1.25(0.96-1.61)	0.97(0.56-1.69)
High BP	1.16(0.93-1.45)	0.94(0.67-1.31)	1.21(0.91-1.62)	1.09(0.76-1.56)
High FBS	1.13(0.85-1.50)	1.10(0.86-1.42)	0.87(0.57-1.31)	0.97(0.74-1.28)
Low HDL	1.02(0.85-1.22)	0.93(0.46-1.89)	1.02(0.77-1.35)	0.88(0.54-1.42)
Total**				
High TG	1.00(0.87-1.15)	0.93(0.64-1.36)	1.24(0.98-1.55)	0.98(0.59-1.63)
High BP	**1.17(1.00-1.37)**	1.23(0.96-1.56)	1.17(0.90-1.51)	1.13(0.81-1.57)
High FBS	1.05(0.86-1.29)	1.03(0.85-1.24)	1.10(0.79-1.53)	0.96(0.75-1.23)
Low HDL	1.09(0.96-1.24)	0.87(0.59-1.26)	0.84(0.66-1.06)	0.84(0.56-1.28)

Footnote: * adjusted for age and smoking. **adjusted for age, gender, and smoking.

Supplementary Table S5: Hazard Ratios (HR) for Chronic Kidney Disease Incidence Considering Metabolic Health Components and Covariates in the 20-Year Follow-Up of the Tehran Lipid and Glucose Study

	**HR***	**95 % CI**	**P value**
High TG, (Ref: No)	1.13	(1.03 - 1.25)	0.011
High BP, (Ref: No)	1.28	(1.17 - 1.41)	<0.001
High FBS, (Ref: No)	1.16	(1.05 - 1.28)	0.004
Low HDL, (Ref: No)	1.08	(0.98 - 1.20)	0.128
Age, (per 1 yr.)	1.09	(1.08 - 1.09)	<0.001
BMI, (Ref: BMI <30 kg/m^2)	1.20	(1.09 - 1.33)	<0.001
Smoking, (Ref: Never)	0.81	(0.70 - 0.94)	0.006
Education, (Ref: Primary)	0.97	(0.88 - 1.07)	0.548
Physical activity, (Ref: low)	0.87	(0.79 - 0.95)	0.002

Footnote: * adjusted for other factors in the table and gender. Ref: Reference group; HR: hazard ratio; CT: confidence interval; TG: triglyceride; BP: blood pressure; FSB: fasting blood glucose; HDL: high density lipid; BMI: body mass index.

Supplementary Table S6: Impact of Obesity-Metabolic Health on Chronic Kidney Disease (CKD), Stratified by Number of Metabolic Health Components

	person-years	CKD Case	Incidence rate (Per 1000 PYs)	Crude Model	Age-Sex Adjusted Model	Fully adjusted Model a
				HR (95% CI)	HR (95% CI)	HR (95% CI)
**No Obesity**						
0 component	17288	175	10.1 (8.7, 11.7)	1 (Reference)	1 (Reference)	1 (Reference)
1 component	34287	340	9.9 (8.9, 11.0)	0.98 (0.82-1.18)	0.96 (0.80-1.16)	0.97(0.81-1.16)
2 components	24631	430	17.4 (15.9, 19.2)	1.76 (1.48-2.10)	1.17 (0.98-1.40)	1.19(0.99-1.41)
3 components	11179	334	29.8 (26.8, 33.2)	3.25 (2.70-3.90)	1.47 (1.22-1.77)	1.48(1.23-1.78)
4 components	2740	124	45.2 (37.9, 53.9)	5.33 (4.23-6.71)	1.60 (1.27-2.03)	1.64(1.30-2.07)
**Obesity**						
0 component	1390	23	16.5(11.0-24.9)	1 (Reference)	1 (Reference)	1 (Reference)
1 component	5935	101	17.0(14.0-20.7)	1.01 (0.64-1.58)	1.02 (0.65-1.60)	1.02 (0.65-1.60)
2 components	8376	178	21.3(18.3-24.6)	1.28 (0.83-1.98)	1.23 (0.80-1.90)	1.25 (0.81-1.93)
3 components	5887	181	30.7(26.6-35.6)	1.91 (1.24-2.95)	1.34 (0.86-2.06)	1.33 (0.86-2.05)
4 components	2592	98	37.8(31.0-46.1)	2.52 (1.60-3.97)	1.36 (0.86-2.14)	1.39 (0.88-2.20)

Footnote: Model 1: crude rate (no adjustment); [a] Model 2: adjusted for age and sex; Model 3: adjusted for age, sex, smoking, education, and physical activity.

Supplementary Table S7: Concordance of Waist Circumference (WC) and Obesity (Defined by Body Mass Index) with Chronic Kidney Disease (CKD) in the Context of Metabolic Health

	person-years	CKD Case	Incidence rate (Per 1000 PYs)	Crude Model	Age-Sex Adjusted Model	Fully adjusted Model a
				HR (95% CI)	HR (95% CI)	HR (95% CI)
**Male**						
MH-NlWC	25184.474	262	10.4(9.2-11.7)	1 (Reference)	1 (Reference)	1 (Reference)
MU-NlWC	3188.1697	79	24.8(19.9-30.9)	2.50(1.94-3.22)	1.82(1.42-2.35)	1.83(1.42-2.35)
MH-AbWC	15338.16	212	13.8(12.1-15.8)	1.43(1.19-1.71)	1.09(0.91-1.31)	1.11(0.93-1.33)
MU-AbWC	7566.8446	181	23.9(20.7-27.7)	2.60(2.15-3.14)	1.54(1.28-1.87)	1.57(1.29-1.90)
**Female**						
MH-NlWC	36470.059	434	11.9(10.8-13.1)	1 (Reference)	1 (Reference)	1 (Reference)
MU-NlWC	3934.7378	148	37.6(32.0-44.2)	3.45(2.86-4.15)	1.50(1.24-1.82)	1.51(1.25-1.83)
MH-AbWC	14493.164	335	23.1(20.8-25.7)	2.09(1.81-2.41)	1.10(0.95-1.28)	1.12(0.97-1.30)
MU-AbWC	7670.4422	328	42.8(38.4-47.6)	4.19(3.63-4.84)	1.25(1.07-1.47)	1.28(1.09-1.50)
**Total**						
MH-NlWC	61654.532	696	11.3(10.5-12.2)	1 (Reference)	1 (Reference)	1 (Reference)
MU-NlWC	7122.9076	227	31.9(28.0-36.3)	3.03(2.61-3.52)	1.71(1.47-1.99)	1.68(1.44-1.95)
MH-AbWC	29831.324	547	18.3(16.9-19.9)	1.75(1.56-1.95)	1.10(0.98-1.23)	1.09(0.97-1.22)
MU-AbWC	15237.287	509	33.4(30.6-36.4)	3.40(3.03-3.81)	1.45(1.29-1.63)	1.43(1.27-1.61)

Footnote: MH-NlWC: metabolically healthy - normal waist circumference; MU-NlWC: metabolically unhealthy - normal waist circumference; MH-AbWC: metabolically healthy – abnormal waist circumference; MU-AbWC: metabolically unhealthy - abnormal waist circumference. Model 1: crude rate (no adjustment); a Model 2: adjusted for age and sex; Model 3: adjusted for age, sex, smoking, education, and physical activity.

Refrences

1. Sarafidis PA, Ruilope LM. Insulin resistance, hyperinsulinemia, and renal injury: mechanisms and implications. Am J Nephrol. 2006;26(3):232-44. DOI: 10.1159/000093632.
2. Yang S, Cao C, Deng T, Zhou Z. Obesity-Related Glomerulopathy: A Latent Change in Obesity Requiring More Attention. Kidney and Blood Pressure Research. 2020;45(4):510-22. DOI: 10.1159/000507784.
3. Lin L, Tan W, Pan X, Tian E, Wu Z, Yang J. Metabolic Syndrome-Related Kidney Injury: A Review and Update. Front Endocrinol (Lausanne). 2022;13:904001. DOI: 10.3389/fendo.2022.904001.
4. Tanner RM, Brown TM, Muntner P. Epidemiology of Obesity, the Metabolic Syndrome, and Chronic Kidney Disease. Current Hypertension Reports. 2012;14(2):152-9. DOI: 10.1007/s11906-012-0254-y.
